# How Catholic Health Systems Made “Conscientious Objection” Unconscionable

**DOI:** 10.1017/jme.2025.45

**Published:** 2025

**Authors:** Lori Freedman, Maryani Palupy Rasidjan

**Affiliations:** 1 ANSIRH/Bixby/Ob-Gyn, University of California San Francisco, Oakland, California, United States; 2 Anthropology, New York University, New York, New York, United States

**Keywords:** conscience, conscientious objection, abortion, religious health systems

## Abstract

In response to “Origin of ‘Conscientious Objection’ in Health Care: How Care Denials Became Enshrined into Law Because of Abortion,” in which Christian Fiala, Joyce Arthur, and Amelia Martzke trace the origins of “conscientious objection” (CO) policy, this commentary looks at the implications of their arguments for large religious health systems where CO disingenuously constrains care. Within such health institutions, the constraints on standard obstetric care reflect the conscience of bishops who write religious policy, not the beliefs of providers who must implement them, or the patients subject to them.

*“There were two members of the committee who were very vocally sort of accusing us of carrying out an elective abortion. And I said, you know, “There was nothing elective about this. This woman didn’t choose to have her membranes rupture at nineteen weeks. She didn’t choose to have a baby with the most severe form of congenital heart disease. There was nothing elective about this.”* ^1^

The physician speaking above is an obstetrician-gynecologist who worked in a Catholic hospital where, per the institution’s religious policy, abortion is prohibited.^2^ In a confidential interview she recounted her experience defending herself to the hospital’s Ethics Committee as they reviewed her team’s decision to induce labor in a patient admitted with previable premature rupture of membranes and multiple fetal anomalies. The physician’s conscience moved her to provide this obstetric intervention before the patient became infected and medically unstable, i.e. before the abortion was theologically allowable per the religious directives that govern care in Catholic hospitals. This practice of delay and denial has led to bad outcomes and occasionally deaths in Catholic hospitals and in US states with abortion bans.^3^ Regardless, this doctor endured humiliation and threats from the hospital’s religious leadership as she was reprimanded. Per US law, institutions have “conscience rights” that can supersede those of their employees and patients. That is, even when abortion is the safest treatment for an admitted patient during an obstetric loss, clinicians must subjugate their medical expertise to the “conscientious objection” of their institution of employment, and are threatened with loss of hospitals privileges or employment if they do not.

In “Origin of ‘Conscientious Objection’ in Health Care: How Care Denials Became Enshrined into Law Because of Abortion,” Christian Fiala, Joyce Arthur, and Amelia Martzke trace the origins of “conscientious objection” (henceforth CO) policy.^4^ The UK was the first country to include one in their abortion law, in 1967, which paved the way for countries from all continents to follow suit. CO in many countries is used to protect individual providers from professional and legal liability if they refuse abortion to patients, and in some cases, to protect them even when refusing to help patients locate alternate providers. The authors argue that the basis for CO is unprincipled, and its inclusion was “fundamentally rooted in opposition to the self-determination of women”; more of a political compromise with Catholic powers than an ethically justifiable law. Going further, they argue that the only legitimate and patient-centered acts of conscience in medicine include (1) “conscientious commitment” which is provision of needed care despite unjust policies or barriers; much like the doctor speaking above, (2) refusal to provide questionable treatments without genuine patient consent, and (3) refusal to provide treatments that are not beneficial to the patient (p. 97). The last, in particular, might be contested by those who oppose abortion, but that’s precisely their point in renaming CO as “belief-based refusals.” If abortion is legal, and the patient deems it beneficial to their life for any reason (medical, social/emotional, economic), the physician’s belief to the contrary, they argue, is physician-centered, not patient-centered.

The power of the CO framing is that it was immediately salient and sympathetic to the mid-20^th^ century public, connoting a powerless subject’s assertion of their morality in the face of evil, as it was for military conscripts who could not or would not kill.^5^ In war, only deeply religious and morally committed people could claim CO, at some cost to themselves, to avoid military duty or at least combat. The authors of the article decry this equation of belief-based refusals of health care with this military precedent of CO. They deem it a farce and one that has been deliberately employed to constrain women’s (and increasingly transgender people’s) autonomy and self-determination.

They make a compelling argument that such belief-based refusers should instead choose a different medical specialty, if they cannot provide patients all their legal and standard choices of reproductive care. They are not the first to make this argument by any means,^6^ and despite the argument lacking legs in law and policy historically, the authors have elevated it, challenging the disingenuousness of many CO claims. They point out: CO is meant to protect doctors who already have power, choice, options. What about patients, many of whom have none?

We would add that CO is more disingenuous than most know in the United States where it is employed by the US Bishops who author the reproductive policies of giant corporate health systems and require their enforcement.^7^ Catholic hospitals treat about 1 in 7 patients^8^ in the US. And in a growing number of states, the majority of their obstetric patients — in some cases as much as 80 percent — are women of color.^9^ Catholic hospitals use public funding and for the most part operate like all their competitors. Yet, unlike their competitors, by claiming “institutional conscience protections,” they are able to mandate that clinicians working in their facilities delay and deny abortion and related care as a condition of employment and privileges. For many people, Catholic hospitals are the only hospitals they can access,^10^ and 37% are not even aware of the religious affiliation when their own hospital is Catholic,^11^ as many hospitals offer little information as to their religious boundedness.^12^

Undergirding CO is the notion that Catholic providers and institutions have the right to force their providers to deny care. Unbeknownst to their patients, the potential precarity of their medical condition is governed and constrained by a religious mandate that they are (1) unaware of and (2) may object to. This kind of invisible religious governance creates real health care barriers and can lead to deadly consequences.^13^ In the clinical encounter is the patient, the provider, and the Catholic Church. While individual belief-based refusers are protected by CO laws, providers who conscientiously object to their Catholic institutions reproductive policies and mandates have no protections, and their patients less so. Providers stand to lose their employment without any recourse to fight for their jobs and keep treating their patients.

Furthermore, because providers working within Catholic institutions for the most part do not share the beliefs that the bishops enshrine in hospital policies, and because they want to prevent harm to their patients, they regularly depend on others to do what is not allowed there to protect patients lives.^14^ Doctors deny care because their employer makes them, then they punt patients to other health care facilities for the necessary care. In utter convolution, the bishops use the CO law to require health care providers with different beliefs to sublimate their medical expertise and training and abide the bishops’ beliefs. Left being bounced around between health facilities, with each hour, car or bus ride, phone call and new clinical encounter to arrange, are the pregnant individuals desperate for the medical care they need and deserve. If individual CO is unprincipled, institutional CO is unconscionable.
